# Impact of Salmonid alphavirus infection in diploid and triploid Atlantic salmon (*Salmo salar* L.) fry

**DOI:** 10.1371/journal.pone.0179192

**Published:** 2017-09-26

**Authors:** Tharangani K. Herath, Angela J. Ashby, Nilantha S. Jayasuriya, James E. Bron, John F. Taylor, Alexandra Adams, Randolph H. Richards, Manfred Weidmann, Hugh W. Ferguson, John B. Taggart, Herve Migaud, Mark J. Fordyce, Kim D. Thompson

**Affiliations:** 1 Institute of Aquaculture, University of Stirling, Stirling, United Kingdom; 2 Department of Animal Production, Welfare and Veterinary Sciences, Harper Adams University, Newport, Shropshire, United Kingdom; 3 Fish Vet Group, Inverness, Scotland, United Kingdom; 4 Marine Medicine Programme, School of Veterinary Medicine, St. George’s University, Grenada, West Indies; 5 Marine Scotland Science, Aberdeen, United Kingdom; 6 Moredun Research Institute, Edinburgh, United Kingdom; National Cheng Kung University, TAIWAN

## Abstract

With increasing interest in the use of triploid salmon in commercial aquaculture, gaining an understanding of how economically important pathogens affect triploid stocks is important. To compare the susceptibility of diploid and triploid Atlantic salmon (*Salmo salar* L.) to viral pathogens, fry were experimentally infected with Salmonid alphavirus sub-type 1 (SAV1), the aetiological agent of pancreas disease (PD) affecting Atlantic salmon aquaculture in Europe. Three groups of fry were exposed to the virus via different routes of infection: intraperitoneal injection (IP), bath immersion, or cohabitation (co-hab) and untreated fry were used as a control group. Mortalities commenced in the co-hab challenged diploid and triploid fish from 11 days post infection (dpi), and the experiment was terminated at 17 dpi. Both diploid and triploid IP challenged groups had similar levels of cumulative mortality at the end of the experimental period (41.1% and 38.9% respectively), and these were significantly higher (p < 0.01) than for the other challenge routes. A TaqMan-based quantitative PCR was used to assess SAV load in the heart, a main target organ of the virus, and also liver, which does not normally display any pathological changes during clinical infections, but exhibited severe degenerative lesions in the present study. The median viral RNA copy number was higher in diploid fish compared to triploid fish in both the heart and the liver of all three challenged groups. However, a significant statistical difference (p < 0.05) was only apparent in the liver of the co-hab groups. Diploid fry also displayed significantly higher levels of pancreatic and myocardial degeneration than triploids. This study showed that both diploid and triploid fry are susceptible to experimental SAV1 infection. The lower virus load seen in the triploids compared to the diploids may possibly be related to differences in cell metabolism between the two groups, however, further investigation is necessary to confirm this and also to assess the outcome of PD outbreaks in other developmental stages of the fish when maintained in commercial production systems.

## Introduction

Farmed Atlantic salmon (*Salmo salar*, Linnaeus, 1758) production has increased rapidly during the last decade, and as the industry grows there is a rising consumer pressure to minimise the environmental and ecological impacts of farming operations. One particular concern comes from fish escapees potentially polluting the gene pool of wild salmon populations. Thus, the use of sterile triploid stock has gained increasing interest in recent years, with additional benefits of sterility, improved flesh quality and better growth through lack of sexual maturation prior to harvest [1,2]. Furthermore, the potential for higher growth rates in triploid salmon may translate into shorter production times, thus reducing exposure to disease [3,4]. However, it has generally been perceived within the salmon production industry that triploid fish are more susceptible to disease, more prone to physical deformities and less able to deal with sub-optimal environmental conditions [5,6]. Moreover, studies in other salmonid species have shown that triploids may be more susceptible to bacterial infections than diploids. For example, triploid coho salmon (*Oncorhynchus kisutch* (Walbaum 1792)) were reported to be more susceptible to vibriosis, while triploid rainbow trout (*Oncorhynchus mykiss*, (Walbaum, 1792)) appeared to be more prone to bacterial gill disease [7]. In order for triploid Atlantic salmon to be considered as a viable option for commercial production, it is necessary to establish if their response to disease challenge is at least comparable to, or better than that of their diploid counterparts.

Viruses from the genus *Alphavirus* (family *Togaviridae*) are known to cause disease in a range of vertebrate hosts [8,9]. Salmon alphavirus (SAV) is a relatively recently characterised species of alphavirus, with six geographically and genotypically distinct subtypes having been isolated from salmonids since 1995 [10,11]. SAV is the aetiological agent behind two economically devastating diseases affecting European salmonid aquaculture: pancreas disease (PD) in Atlantic salmon and sleeping disease (SD) in rainbow trout [12,13]. Clinical PD generally occurs in the sea water phase of the production cycle, leading to mortality levels ranging between 1–50% [14,15]. PD poses a significant welfare concern for salmon producers in Europe [14,16] with reduced feed intake and subsequent loss of growth following infection [17]. In 2003–2004 it was estimated that PD resulted in the loss of up to 30% of the stock in a number of Irish farms, translating into an operating loss of €12M across the Irish salmon industry [18]. In 2012, Larsson *et al*. [19] reported that an average of 90 sites were diagnosed with PD each year in Norway since 2006, with losses estimated at ~€1.8M for each farm affected. A review of farm records from one of Scotland’s largest salmon producers revealed that from 2000–2009, PD accounted for the loss of 8.6% of total biomass, which was higher than for any other infectious disease [20].

The clinical progression of natural PD infection occurring in the sea water phase of the production cycle is typically characterised by three histologically distinct phases [21]. The initial acute phase lasts for up to 10 days at 2–14°C, during which time infected fish may exhibit external signs such as lethargy, inappetence and production of yellow faecal casts due to lack of feeding. During this phase, inflammation in the pancreas and heart are also histologically detectable. The sub-acute phase lasts around 10–21 days from the onset of clinical signs and is characterised by histological lesions in pancreatic tissue, heart and skeletal muscle. Skeletal muscle lesions are the predominant histological feature observed in the chronic stages of infection, usually lasting for up to 42 days.

The aim of the present study was to evaluate the effects of an SAV1 infection in triploid Atlantic salmon fry relative to diploid fry, comparing their susceptibility to SAV1 infection and the disease pathogenesis that resulted using different routes of infection.

## Materials and methods

### Fish

This study was carried out in accordance to the UK (Scientific procedures) Act 1986 at Institute of Aquaculture, University of Stirling, UK (Ethical approval was given by Institute of Aquaculture, University of Stirling Ethics Committee under project licence PLL 60/4189). Fish anesthesia was performed using for benzocaine (40 mg L-1) (Sigma-Aldrich, Dorset, England) to inject 0.05 mL of virus intraperitoneally and fish were sacrificed using overdose benzocaine 100 mg/mL).

Full-sib groups of diploid and triploid Atlantic salmon eggs (IPNV-QTL resistant strain) were supplied from a commercial breeding company to Howietoun Fish Farm, Stirling (56°N, 4°W) at 395 degree-days post-fertilisation (^○^DPF). Triploidy was induced using a hydrostatic pressure shock of 655 bar for 6.25 min at 8°C, applied 37 min post-fertilisation. From fertilisation to the point of shipping to the fish farm, the ova were incubated at 6.0 ±0.5°C. Ova were reared under constant darkness at 8.7 ±1.0°C until the start of first feeding, (929^○^DPF; March 26, 2012), after which they were then reared communally under constant light. To verify their ploidy status, smears were prepared from blood collected following severing of the caudal peduncle of euthanized 1.5 g fish (n = 100/ploidy). After air drying, slides were fixed in 100% methanol and then placed into Giemsa stain for 10 min. The diameter of the nuclei of erythrocytes were measured from images captured under 100× oil immersion objective using image analysis software (ImagePro). A total of 20 randomly chosen nuclei per slide were measured to the nearest 0.01 μm. The diameter of erythrocyte nuclei of the diploid groups were significantly smaller than nuclei from pressure shock triploid groups (2N 6.9–7.8 μm; 3N 9.1–10.2 μm), confirming that all fish subjected to hydrostatic pressure shock were likely to be triploids.

Fry of each ploidy were transferred to the Aquatic Research Facility, Institute of Aquaculture (IoA), University of Stirling. From the receipt of eggs until the trial commencement, mortality was recorded as 6.5% for diploids and 9.1% for triploids.The photoperiod in the rearing facility was maintained on a 12:12 h light and dark cycle, and fry were fed *ad libitum* twice a day throughout the experiment using a commercial salmon feed (Nutra-parr, EWOS). Prior to performing the infection experiment, a cohort of fish were screened by cell culture to confirm that they were free of common salmonid viral diseases.

### Preparation of stock virus for challenge experiment

An Irish salmon alphavirus subtype 1 (SAV1) isolate (F02-143), originating from an SAV1 outbreak in Ireland in 2002 (kindly provided by Dr David Graham, Agri-Food and Bioscience Institute, Belfast, Northern Ireland) was used for the experimental infection. Stock virus was grown in Chinook salmon embryo (CHSE)-214 cells, (Passage 9), and had a 50% tissue culture infective dose (TCID_50_) of 10 ^8.166^ mL^-1^.

### Tank set up and challenge experiment

The experimental challenge was conducted in clear, rectangular 10 L tanks, supplied with dechlorinated flow-through water. The maximum flow rate of the system was 500 mL min^-1^ and temperature was maintained at 10 ±1°C. Diploid and triploid fry, with an average weight of 1.8 g ±0. 9 and 1.6 ±1.3 g respectively, were randomly allocated into 12 tanks per ploidy (n = 30 fry/tank/ploidy), were infected with SAV1 using either an immersion, intraperitoneal (IP) injection or co-habitation (co-hab) route of infection, with the fourth group left unchallenged as controls. Each group was held in triplicate tanks.

The group challenged by immersion was placed in a 2 L bath containing SAV1 at a titre of 5 x 10^4^ mL^-1^ TCID_50_ for 2 h with aeration. The group infected with the virus by IP injection were anaesthestised with benzocaine (40 mg L^-1^) (Sigma-Aldrich, Dorset, England) and injected with 50 μL of the virus (TCID_50_ mL^-1^ = 10^5^) using an insulin syringe with a 27 gague needle. For the co-hab challenge, three fry were injected IP as described above and placed into a tank containing thirty naïve fry. The infected fry were fin-clipped to facilitate identification. The fish were monitored four times daily for changes in their behaviour, appearance, morbidity and mortality throughout the experimental period. Dead and moribund fish (unable to maintain their balance and swimming erratically at the water surface) were removed and moribund fosh were killed with an overdose of benzocaine (100 mg L^-1^).

### Sampling

The experiment was terminated at 17 days post infection (dpi). At the time of termination, the remaining experimental fish were euthanized with an overdose of benzocaine. A longitudinal ventral midline incision was made to facilitate fixative penetration before fixing fry in either RNA stabilisation buffer (3.6 M ammonium sulphate, 18 mM sodium citrate, 15 mM EDTA, pH 5.2), or 4% DNAse-/RNAse-free neutral buffered paraformaldehyde (NBPF). After 24 h, the RNA stabilisation buffer was removed and samples stored at -70°C. The NBPF fixed fish were transferred into 75% ethanol and stored at 4°C until processing for routine histology. Survival data from the experiment were analysed using Kaplan-Meier life-table analysis in IBM SPSS^®^ version 19.

### Virus load in tissues

#### RNA isolation

The whole heart and a piece of liver tissue (1–2 mm^3^) from five fry from each tank were placed into separate pre-weighed tubes filled with TriReagent^®^ (Sigma Aldrich, Dorset, UK). The weight of the tissues was determined before homogenising the samples using a Fastprep^®^ (Oxford Biosystems, Milton Park, UK) tissue homogeniser. The total RNA from heart and liver homogenates was then extracted following manufacturer’s instructions for TriReagent^®^ RNA extraction. Finally, ethanol-washed RNA pellets were dissolved in nuclease free (DNAse-/RNAse-free) water before storing at -70°C until analysed.

#### RT-qPCR assay for heart and liver tissue

To develop an RNA standard for the quantitative reverse transcription real time PCR (qRT-PCR)) assay, a target amplicon targeting the non-structural protein 1 (nSP1) of SAV-1 [22] was generated and ligated into a pCR^®^II plasmid using a TA cloning^®^ kit, with PCR II Vector and One Shot Top10F’ chemically competent *E coli* (Life Technologies, Paisley, UK) following manufacturer´s instructions. To prepare the DNA template for molecular cloning, viral RNA was extracted from SAV culture supernatant using a NucleoSpin^®^ RNA Virus isolation kit (Macherey-Nagel, Fisher Scientific, Leicestershire, UK). The viral RNA was then reverse transcribed using a High-Capacity cDNA Reverse Transcription Kit (Applied Biosystems, Thermo Fisher Scientific, Leicestershire, UK). The total RNA was PCR amplified using a 108 bp long primer pair (nSP1-F ‘CCGGCCCTGAACCAGTT3’ and nSP1-R ‘GTAGCCAAGTGGGAGAAAGCT3’) to prepare the DNA template for ligation into the pCR^®^II Vector with a Dual Promoter (Invitrogen, Thermo Fisher Scientific, Leicestershire, UK). The recombinant plasmid DNA was extracted with a Wizard^®^ Plus SV Miniprep DNA purification system (Promega, Southampton, UK). DNA from the clone with sense orientation was then *in vitro* transcribed using a T7 RNA polymerase (Roche, West Sussex, UK) in order to obtain IVT-RNA. The RNA quantity present in the reaction was measured using a Quant-iT^™^ RiboGreen^®^ Assay Kit (Life Technologies) and RNA copy number present in the TVT-RNA was determined as described by Fronhoffs *et al*. [23] before diluting to prepare the standard curve.

The RT-qPCR assay was performed in a Roche LightCycler^®^ 480 instrument using the LightCycler^®^ 480 RNA Master Hydrolysis Probe according to the manufacturer’s instructions. This involved preparing master mix with 500 nM of forward (nSP1-F) and reverse (nSP1-R) primers, 200 nM of MWG probe (5’-AGAGCGCTGACTCGGCAACCGT -3’) [22] and 1 μl of total RNA extracted from the tissue or 1 μl IVT-RNA dilutions. The amplification profile consisted of reverse transcription at 63°C for 3 min, activation at 95°C for 30 sec, 45 cycles of amplification at 95°C and 60°C for 15 sec each and final cooling at 40°C for 40 sec. Each 96-well reaction plate consisted of duplicates of IVT-RNA standard, total RNA extracted from samples, non-template control (NTC) and a positive control. The standered curve was generated from the IVT-RNA with an amplification efficiency range of 0.9–1.01.

The absolute copy number of RNA present in the samples was expressed as RNA copy number per milligram of tissue (RNA copies mg^-1^). The results obtained for each group were then analysed in Minitab Version 17 (Minitab Inc). To determine whether there were differences in the viral RNA copy number mg^-1^ tissue present in the heart and liver samples of the virus-challenged diploid and triploid fry infected through the three different challenge routes, data were pre-tested for normality using the Anderson-Darling test. As the normality of the viral copy number in both the heart and liver were skewed, the viral copies mg^-1^ were log transformed before analysing them with a general linear model (GLM). The significance of difference (p ≤ 0.05) between ploidy in each treatment group was evaluated using a Tukey’s *post-hoc* test.

### Sample processing for histology

Five fish from each tank were selected for histology. The heart and abdominal viscera from the body cavity were carefully removed and a 2–4 mm transverse wedge section was prepared immediately anterior to the dorsal fin to obtain a sample of skeletal muscle, skin and kidney. The tissues were routinely processed for histology and stained with haematoxylin and eosin (H&E) or Periodic acid-Schiff-Alcian blue (PAS-AB).

### Histological evaluation

The colour and staining consistency of H&E and PAS-AB stained sections were assessed using an Olympus BX51 DS light microscope before scanning at 40x magnification with an Olympus dotSlide scanning system at the Marine Scotland laboratory in Aberdeen. A semi-quantitative histopathological scoring system adapted from that used by Christie *et al*. 2007 [24] and modified by Herath *et al*. 2013 [25] was used to evaluate changes in skeletal muscle, heart and pancreatic tissue with regards to both severity and distribution (Table 1). A new scoring system was also incorporated to assess liver changes (Table 1). Scoring was performed blind to avoid any assessor bias. Quantitative assessment was only performed on the control and cohabitation groups, from which a complete set of samples was available from all triplicate tanks.

**Table 1:** Histology scoring system used to assess pathology in sampled tissues.

		Heart		Pancreas		Liver		Skeletal muscle
**Inflammation**	0	Normal or very mild	0	Normal or very mild	0	Normal or very mild	0	Normal or very mild
	1	Mild inflammatory infiltration	1	Mild inflammatory infiltration	1	Mild inflammatory infiltration	1	Mild inflammatory infiltration
	2	Moderate inflammatory infiltration	2	Moderate inflammatory infiltration	2	Moderate inflammatory infiltration	2	Moderate inflammatory infiltration
	3	Marked inflammatory infiltration	3	Marked inflammatory infiltration	3	Marked inflammatory infiltration	3	Marked inflammatory infiltration
**Degeneration**	0	Normal	0	Normal	0	Normal	0	Normal
	1	Focal myocardial degeneration/necrosis	1	Focal acinar cell degeneration and necrosis	1	Focal hepatocyte degeneration/necrosis	1	Focal myocyte degeneration/necrosis
	2	Multifocal mild to moderate myocardial degeneration/necrosis	2	Multifocal mild to moderate acinar cell degeneration/necrosis	2	Multifocal mild to moderate hepatocyte degeneration/necrosis	2	Multifocal mild to moderate myocyte degeneration/necrosis
	3	Marked multifocal myocardial degeneration/necrosis	3	Marked multifocal acinar cell degeneration/necrosis	3	Marked multifocal hepatocyte degeneration/necrosis	3	Marked multifocal myocyte degeneration/necrosis
	4	Severe multifocal myocardial degeneration/necrosis	4	Total absence of acinar cells	4	Severe multifocal myocardial degeneration/necrosis	4	Severe multifocal myocyte degeneration/necrosis
**Fibrosis**	0	Absence of fibrosis	0	Absence of fibrosis	0	Absence of fibrosis	0	Absence of fibrosis
	1	Mild fibrosis	1	Mild fibrosis	1	Mild fibrosis	1	Mild fibrosis
	2	Moderate fibrosis	2	Moderate fibrosis	2	Moderate fibrosis	2	Moderate fibrosis
	3	Sever, widespread fibrosis	3	Sever, widespread fibrosis	3	Sever, widespread fibrosis	3	Sever, widespread fibrosis
**Epicarditis**	0	Normal						
	1	Mild epicarditis						
	2	Moderate epicarditis						
	3	Marked epicarditis						

### Histology data analysis

For analysis of histological scoring results, Kruskal-Wallis analysis was performed to identify differences between ploidies, treatments (cohabitation vs control) and tanks, using R-studio ver. 0.98.501, 2013 and R ver. x64 3.2.0. Where necessary, subsequent *post-hoc* testing to identify significant differences was performed using a Nemenyi test also using R-studio. A cumulative link mixed model (CLLM) and likelihood ratio test (LRT) were then applied to rule out any tank effect on the scoring results and to verify the results of Kruskal-Wallis testing. Principal component analysis (PCA) to identify any tank clustering or whether scores in the pancreas, heart and liver showed any correlation in their variance at a tank level was also performed.

## Results

### Mortality

**Fig 1:**
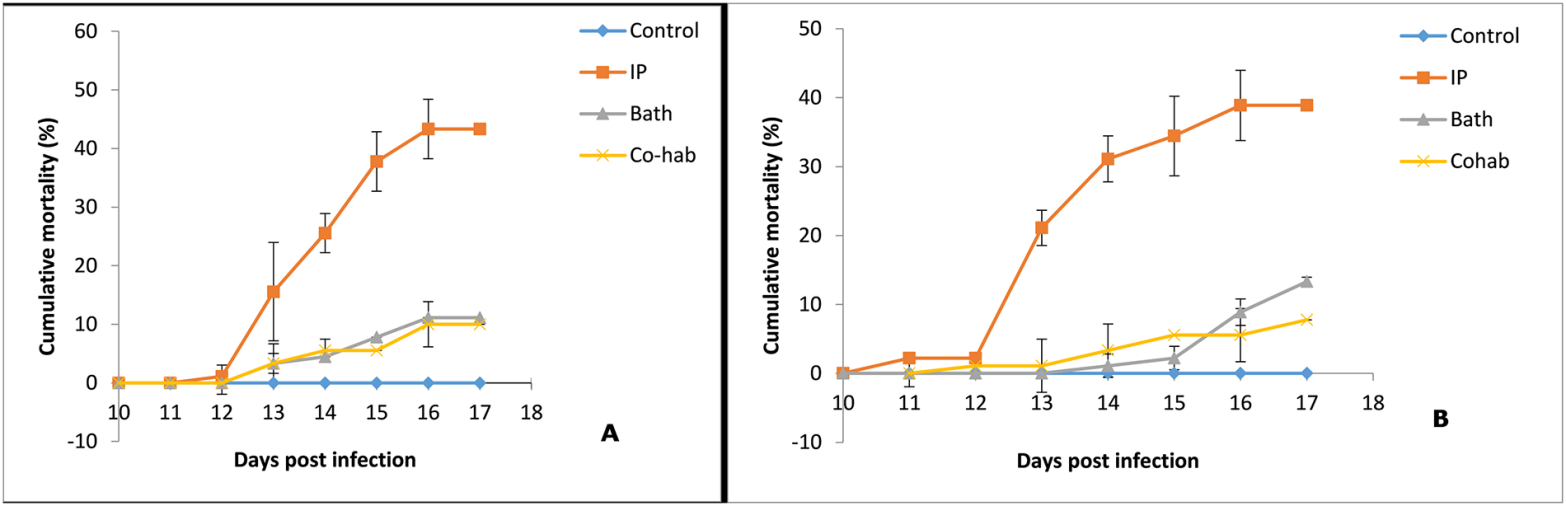
Cumulative daily percentage mortality ± standard deveation of diploid and triploid fry infected with Salmonid alphavirus (SAV). (A) diploid and (B) triploid Atlantic salmon fry (n = 30/tank) exposed to SAV 1, F02-143 Irish isolate via different routes of infection; intraperitoneal (IP) (TCID_50_ = 2.5x10^3^/fry), bath (exposed for 2h for TCID_50_ = 5x10^4^ mL^-1^ x 2L) and co-habitation (three IP injected fish mixed into each tank) compared to untreated control fry.

The daily percentage cumulative mortality for each challenge route was similar between ploidy types throughout the 17 day experimental period (Fig 1).The mortalities in diploid fry started at 12 dpi with one fish dying in the IP group at this time, while co-hab group it was three. At 13 dpi three fish had died in both the immersion group and in the co-habitaion group. The first mortalities satarted to occur in the triploid fry at 11 dpi, with two fish dying in the IP groupand one in the co-hab group. Fish in the immersion group started dying from 14 dpi. No mortalities were observed amongst uninfected control fry over the course of the study.

The mean cumulative mortality across the groups infected with the virus reached 21%. When separated by ploidy, diploid and triploid virus challenged fry experienced 21% and 20.7% mortality, respectively (Kaplan-Meier life-table analysis SPSS p ≥ 0.05). Triploid fish infected by IP injetion had the higher initial mortalities, reaching a peak mortality on 13 dpi (n = 17), compared with the diploid IP injected fry, while mortalities declined faster in the triploid IP injected fry than their diploid counterparts. There was no significant difference between the two groups by the end of the experiment. Both diploid and triploid IP challenged groups had relatively similar cumulative mortalities (p ≥ 0.05) at the end of the experiment (43.3% and 38.9% respectively). It was notable that mortalities in both triploid and diploid IP challenged groups were significantly higher (p ≤ 0.0001) than those for the other challenge groups. However, when the effect of treatment was taken into consideration, one tank of triploid IP injected fry had a significantly lower than expected mortality (p <0.05), with a 10% mortality rate.

### Virus load

The virus load in the heart and liver of experimentally infected and control fry was determined using a TaqMan-based qRT-PCR, for which a plasmid-derived transcript, encoding the nsP1 region of the SAV genome, was *in vitro* transcribed. The stock IVT-RNA for standered curve was adjusted for 5 x10^7^ and the lowest detection limit (cut off) was 5 x10^1^, with samples values above this considered positive.

All fish in control groups tested negative for SAV. From the samples taken from virus-exposed diploid and triploid fry, 86% heart samples and 78.9% of liver samples were found to be positive for SAV. In diploid fry, 91.0% of heart samples and 82.0% of liver samples were positive for the virus, while for triploids, 80% of heart and 75.5% of liver samples were positive. In diploid fry, 100% of heart samples from both the IP and IM challenged groups were positive for SAV, however, only 73.3% were positive in the co-hab group. Amongst the triploid fry, the IM group had the highest percentage of SAV positive hearts (93.3%), followed by the IP and co-hab groups, in which 86.6% and 60% were SAV positive, respectively (Table 2). The percentage of positive livers in triploid fryfor the three challenge routes were IP, 86.6%, IM 83.3% and co-hab 60%.

**Table 2:** RT-qPCR results summary.

Heart										
Route of exposure	**Diploid**					**Triploid**				
	**Viral RNA positive fish**					**Viral RNA positive fish**				
	Tank 1 (n = 5)	Tank 2 (n = 5)	Tank 3 (n = 5)	**Total (n = 15)**	**(%)**	Tank 1 (n = 5)	Tank 2 (n = 5)	Tank 3 (n = 5)	Total (n = 15)	(%)
	tank 1	tank 2	tank 3	2n	%	tank 1	tank 2	tank 3	3n	%
IP	5	5	5	15	100.00	3	5	5	13	86.67
IM	5	5	5	15	100.00	5	4	5	14	93.33
Co-hab	5	5	1	11	73.33	4	5	0	9	60.00
**Liver**										
Route of exposure	**Diploid**					**Triploid**				
	**Viral RNA positive fish**					**Viral RNA positive fish**				
	Tank 1 (n = 5)	Tank 2 (n = 5)	Tank 3 (n = 5)	**Total (n = 15)**	**(%)**	Tank 1 (n = 5)	Tank 2 (n = 5)	Tank 3 (n = 5)	Total (n = 15)	(%)
IP	3	5	5	13	86.67	3	5	5	13	86.67
IM	4	4	5	13	86.67	4	3	5	11	73.33
Co-hab	5	5	1	11	73.33	4	5	0	9	60.00

Number of diploid and triploid fry (heart and liver) positive for Salmonid alphavirus by RT-qPCR 17 days post-exposure to the virus by intraperitoneal injection (IP), immersion (IM) or co-habitation (co-hab)

**Fig 2:**
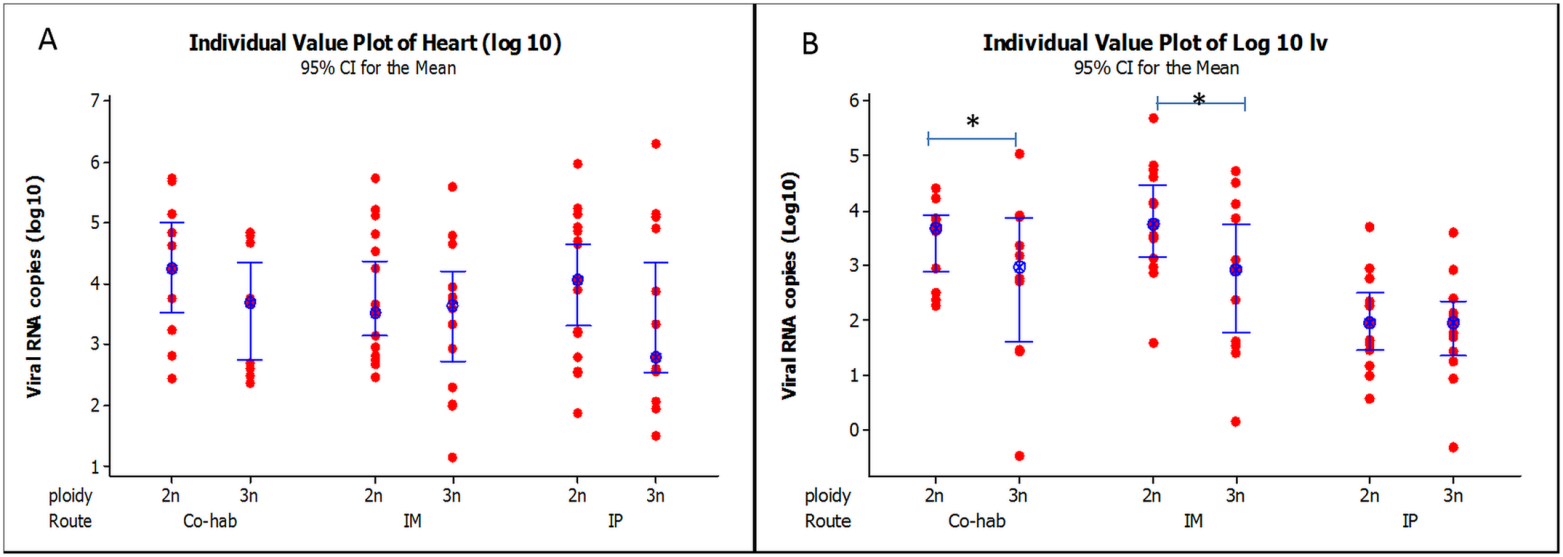
Real time PCR results. Salmonid alphavirus copy number (copies gm tissue^-1^), determined by measuring nSP1 levels by qRT-PCR in triploid and diploid fry infected using either a cohabitation (co-hab), immersion (IM) or intraperioneal (IP) route of infection. No any virus virus was detected in the control fish.

Virus copy number (log_10_) detected in the fry of each group are presented in Fig 2. The heart (Fig 2a) and liver (Fig 2b) tissue samples of diploid fish in each treatment group had higher median SAV RNA copy numbers than their triploid counterparts, and in liver tissues this difference was statistically significant (p ≤ 0.01) for both IP and IM groups. The mean Cp of the RT-qPCR and mean virus copy number for each individual fish is presented in suplementry file S1 Fig.

### Descriptive histopathology

#### Pancreas

**Fig 3:**
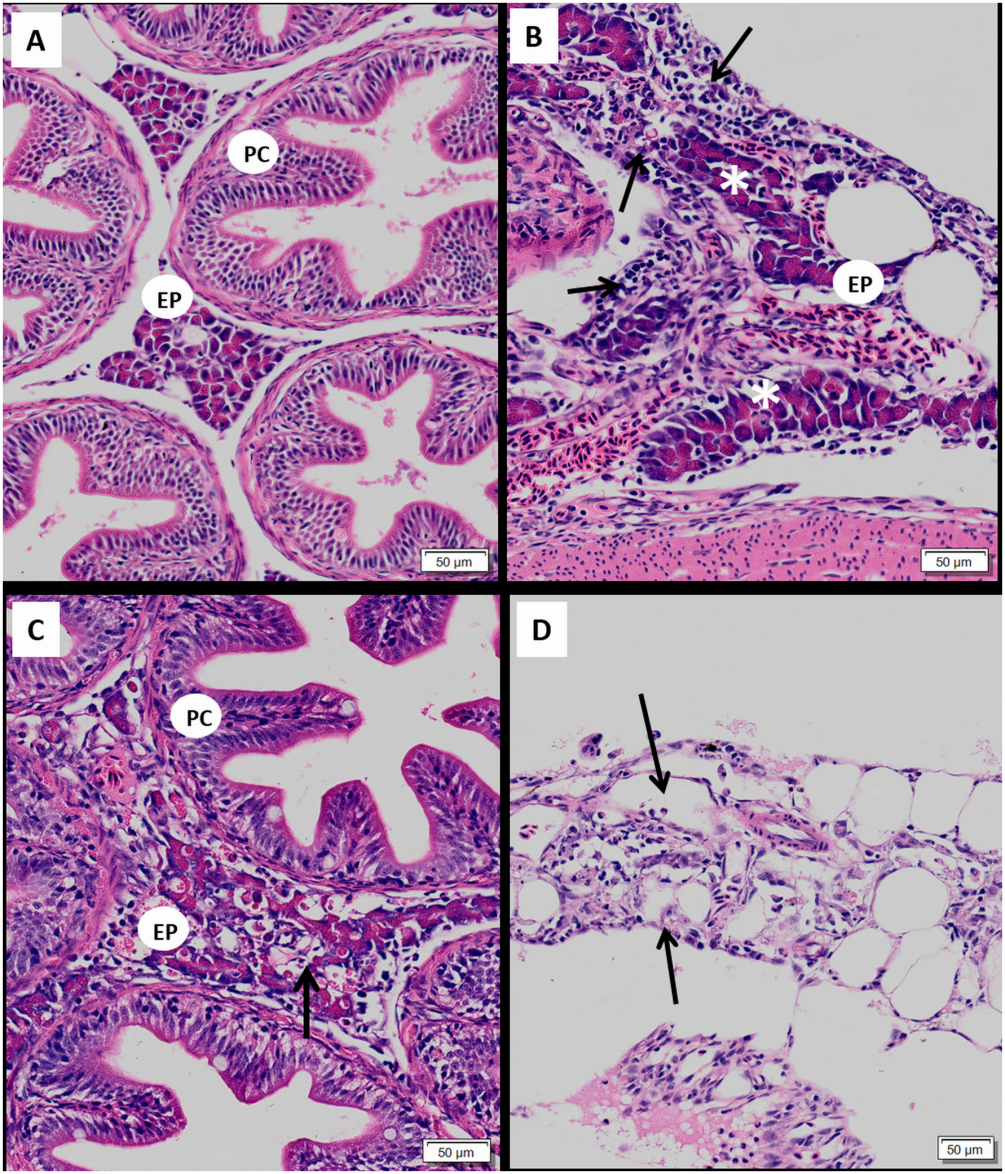
Pancreatic histopathology of SAV-infected Atlantic salmon fry. (A) Normal pancreatic tissue from control fry. Normal exocrine acinar tissue (EP) seen adjacent to a section of pyloric caeca (PC); (B) Exocrine pancreas (EP) with severe diffuse mononuclear inflammatory cell infiltration (arrow) along with some intact acinar cells (*) observed in co-habitation group. (C) Marked acinar necrosis in diploid cohabitation fry, cell breakdown with leakage of zymogen granules (thick arrow) (D) Pancreas from triploid IP fry showing almost complete destruction and absence of acinar tissue (Arrow).

In control fry, pancreatic tissue appeared normal, except for mild focal degeneration observed in a few fry in both diploids (n = 3) and triploids (n = 5) fry. Histological changes characteristic of SAV pathology were observed in pancreatic tissues of both exposed diploid and triploid fry. The degenerative changes observed in the pancreas ranged from mild focal acinar cell degeneration to widespread severe destruction of acinar tissue with complete loss of normal architecture. In some individuals, marked degenerative changes were observed in one area of the exocrine tissue, whilst other areas were largely unaffected. Compared to control fish (Fig 3A), hyper-cellularity of pancreatic tissue due to an influx of mononuclear cells was observed in the challenged fish (Fig 3B). This inflammatory reaction was not always accompanied by concomitant degeneration. Conversely, marked degeneration in the absence of any inflammatory reaction was also observed. Acinar cells were observed in varying stages of degeneration and also necrosis/apoptosis. Rupture of the cell membrane and subsequent leakage of zymogen granules was also noted (Fig 3C and 3D), however, the majority of acinar cells still displayed intact cell membrane and normal nuclear features. Karyorrhexis and cytoplasmic vacuolation of acinar cells accompanied the aforementioned changes in more severely affected individuals. In some fry, little or no acinar tissue remained (Fig 3D). Fibrosis was not observed in any of the pancreas sections examined.

#### Heart

**Fig 4:**
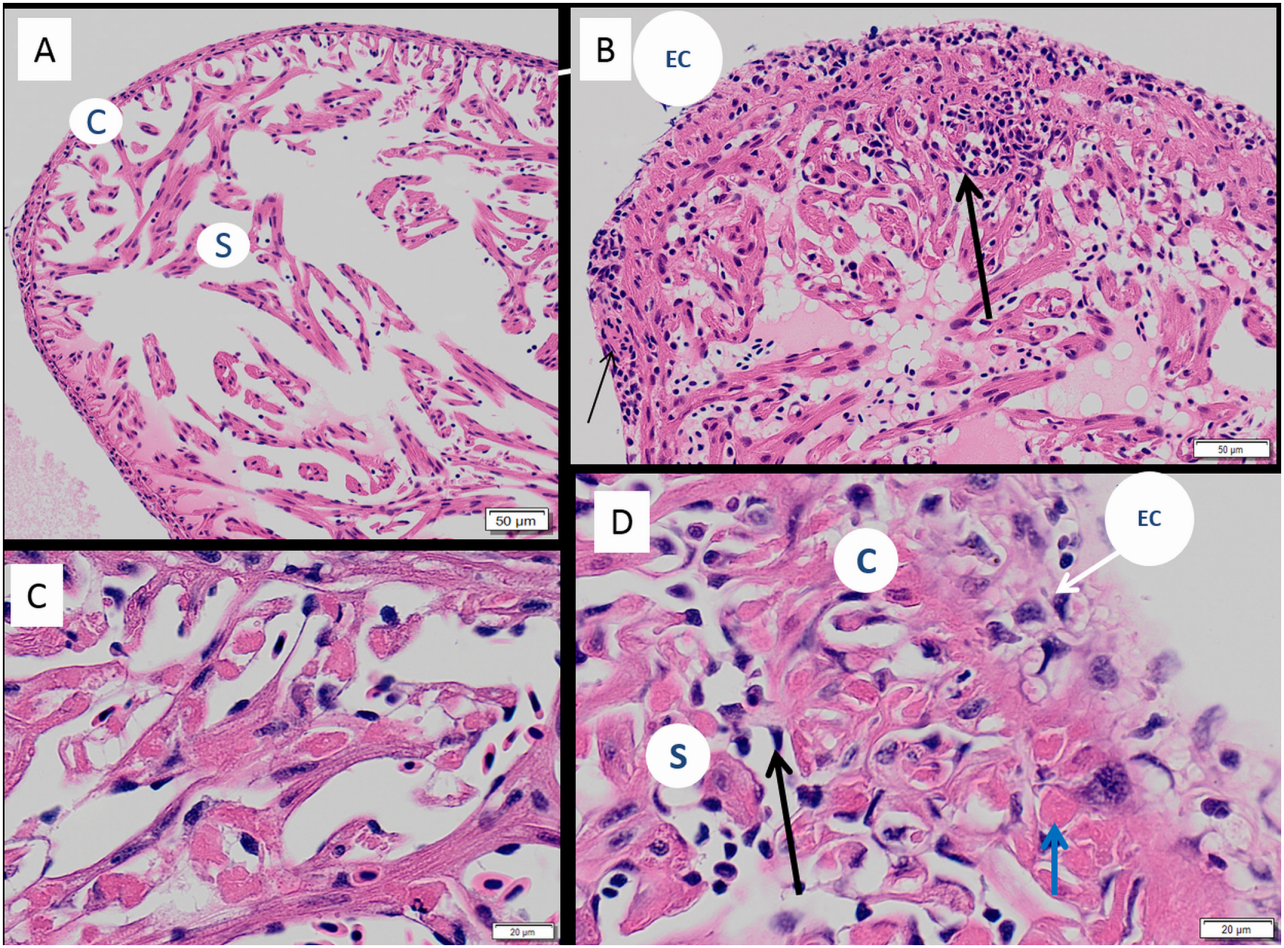
Histopathology of heart in SAV infected Atlantic salmon fry. (A) Normal heart of diploid control fry; (B) Normal heart of diploid cohabitation fry showing cell infiltration in myocardium (thick arrow) and epicardium (thick arrow), increased serous fluid accumulation in the ventricle, and hyper-eosinophilia in the compact and spongy cardiomyocytes; (C) Triploid immersion fry: severe diffuse myocardial degeneration affecting spongy myocardium with hyper-esosinophilia and hyalinisation in myocyte; (D) High magnification view of diploid immersion heart. Inflammatory cell infiltrate within epicardium (EC), spongy myocardium (S) and at interface of the compact spongy myocardium (thick arrow), severe diffuse hyalinisation in compact myocardium (blue arrow), nuclear pyknosis and karyorrhexis.

In the majority of control fry, the ventricle consisted of sparsely distributed spongiosum and a thin compact layer (Fig 4A), however, in one diploid and one triploid fry had mild focal inflammation was observed within ventricles. The histological changes in the heart of infected fish were consistent with SAV1-associated lesions, but, the degree of damage was extensive compared to that which is generally observed in older fish. Across the diploid and triploid fry challenged with the virus by co-hab, both compact and spongy muscle were notably thick, oedematous and hyper-eosinophilic (Fig 4B). Furthermore, a homogenous eosinophilic amorphous material remained in both ventricular and atrial cardiac chambers of severely damaged hearts (Fig 4B). Degeneration in both ventricular spongy and compact myocardium was identified in infected fish. The myocardial degeneration observed was more pronounced than the inflammatory response in the infected fry. The severity of degeneration ranged from mild focal myocyte necrosis to widespread ventricular myocardial necrosis with scant healthy myocardium remaining (Fig 4C). Affected cardiac myocytes displayed loss of striation, pyknotic nuclei, hypereosinophilic cytoplasm and hyaline degeneration, with cells developing a homogenous, glassy appearance (Fig 4C). Profound infiltration of mononuclear inflammatory cells into both spongy and compact myocardium, at the compact and spongy myocardial interface, was seen in some virus exposed fry (Fig 4D). Atrial myocardial necrosis was also observed in some infected fry, but this was not a consistent feature. Mild to moderate focal to widespread epicarditis, consisting of a mixed inflammatory cell origin, was also observed in a number of infected fry (Fig 4D).

#### Skeletal muscle

Most of the diploid and triploid fry from both control and virus exposed groups had no skeletal muscle lesions. However, a small number of virus exposed diploid (n = 2) and triploid (n = 2) fry in the co-hab group, presented mild, focal muscular dystrophy in the red muscle (suplementry data S2 Fig). An inflammatory response was only observed in one triploid fish in the co-hab group.

#### Liver

**Fig 5:**
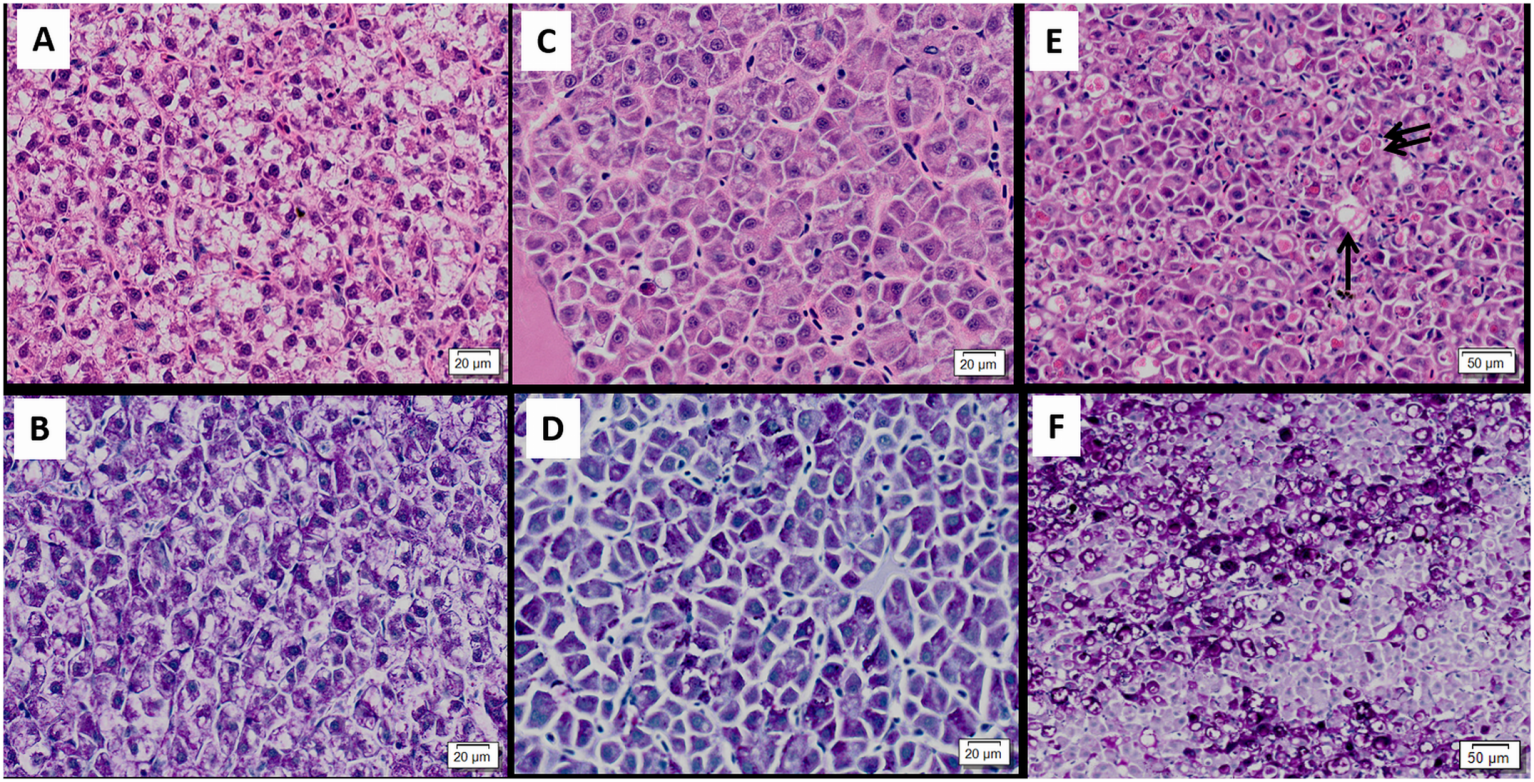
Liver histopathology of SAV infected Atlantic salmon fry. (A) H & E stain and (B) PAS stained liver from diploid control fry showing normal hepatocyte morphology and intracellular lipid with (C) H & E stain and (D) PAS stained liver in diploid cohabitation fry with diffuse moderate degenerative change and depletion of intra-hepatocyte lipid (E) H & E stain and (F) PAS stained of diploid cohabitation fry with severe diffuse coagulative necrosis hypereosinophilia (thick arrow) and cytoplasmic vacuolisation in liver.

Most infected fry displayed a reduced level of intra-cytoplasmic fat in their hepatocytes relative to control fish (Fig 5A). Pathological changes ranged from mildly affected livers with scattered apoptotic or necrotic hepatocytes (Fig 5B) through to diffuse severe coagulative necrosis affecting most of the liver with loss of normal architecture (Fig 5C). The cytoplasm of hepatocytes of control fish (Fig 5D), virus exposed fry showed homogeneous PAS-positive staining (Fig 5E & 5F). Single-cell necrosis was a common feature in diseased livers. In severely affected individuals, the nuclear and cytoplasmic appearance of hepatocytes was highly heterogeneous, with cells in various stages of degeneration and necrosis (Fig 5C & 5F). Damaged and dying hepatocytes were frequently enlarged with the presence of hyper-eosinophilic casts in the cytoplasm (ceroids) (Fig 5C), which became intensely positive with the PAS stain (Fig 5F). The nuclei of these cells were shrunken and showed intensely basophilic staining properties and become intensly positive with periodic acid shiff stain (S3 Fig). In addition, pyknosis, karyorrhexis and karyolysis were also frequently observed, and mild to moderate inflammatory infiltration observed in severely affected fish (S3 Fig). Hepatic congestion with extravasation of erythrocytes was present in some affected individuals. Where present, the inflammatory response observed was mild.

#### Kidney

**Fig 6:**
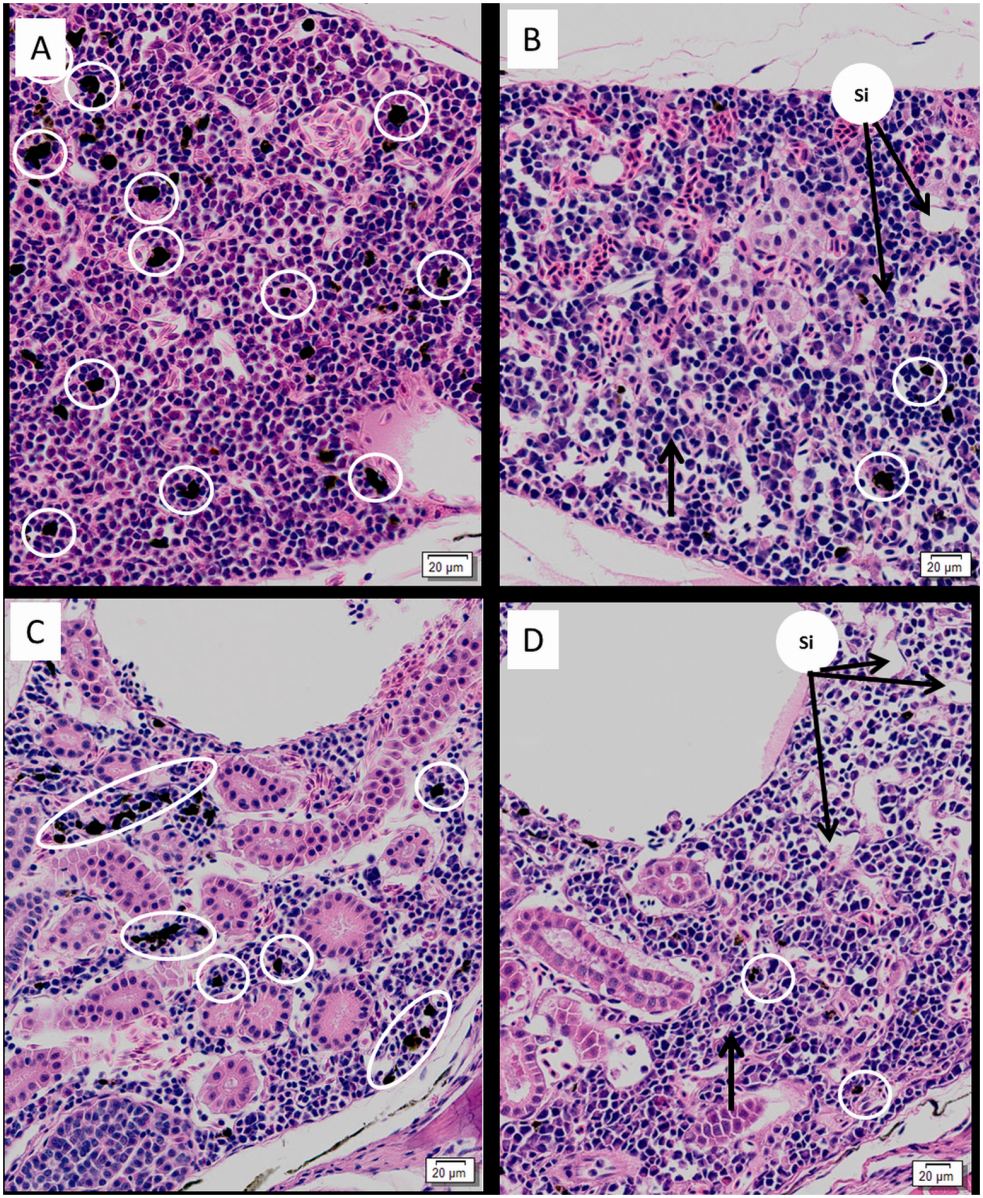
Renal histopathology of SAV infected Atlantic salmon fry. H & E stained (A) trunk kidney; (B) head kidney of unchallenged fish with high number of melanomacrophages (white circles); (C) trunk and (D) head kidney of cohabitation fry, note depletion of melanomacrophages (white circles), clear, enlarged sinusoidal spaces (si) and relatively large parenchymal cells (thick arrow) compared to control fry.

The interstitial tissue of head and trunk kidney appeared depleted, and sinusoids were enlarged in virus-exposed fry compared to controls (Fig 6A–6D). The number of melanomacrophages observed in the interstitial tissue of virus-exposed fry was also markedly depleted (Fig 6B–6D), and haematopoietic cells of the kidney appeared large and contained highly basophilic nuclei (Fig 6B–6D). Renal tubular necrosis was also observed in a number of virus-exposed fry, although this was generally considered mild.

#### Histological scoring

**Fig 7:**
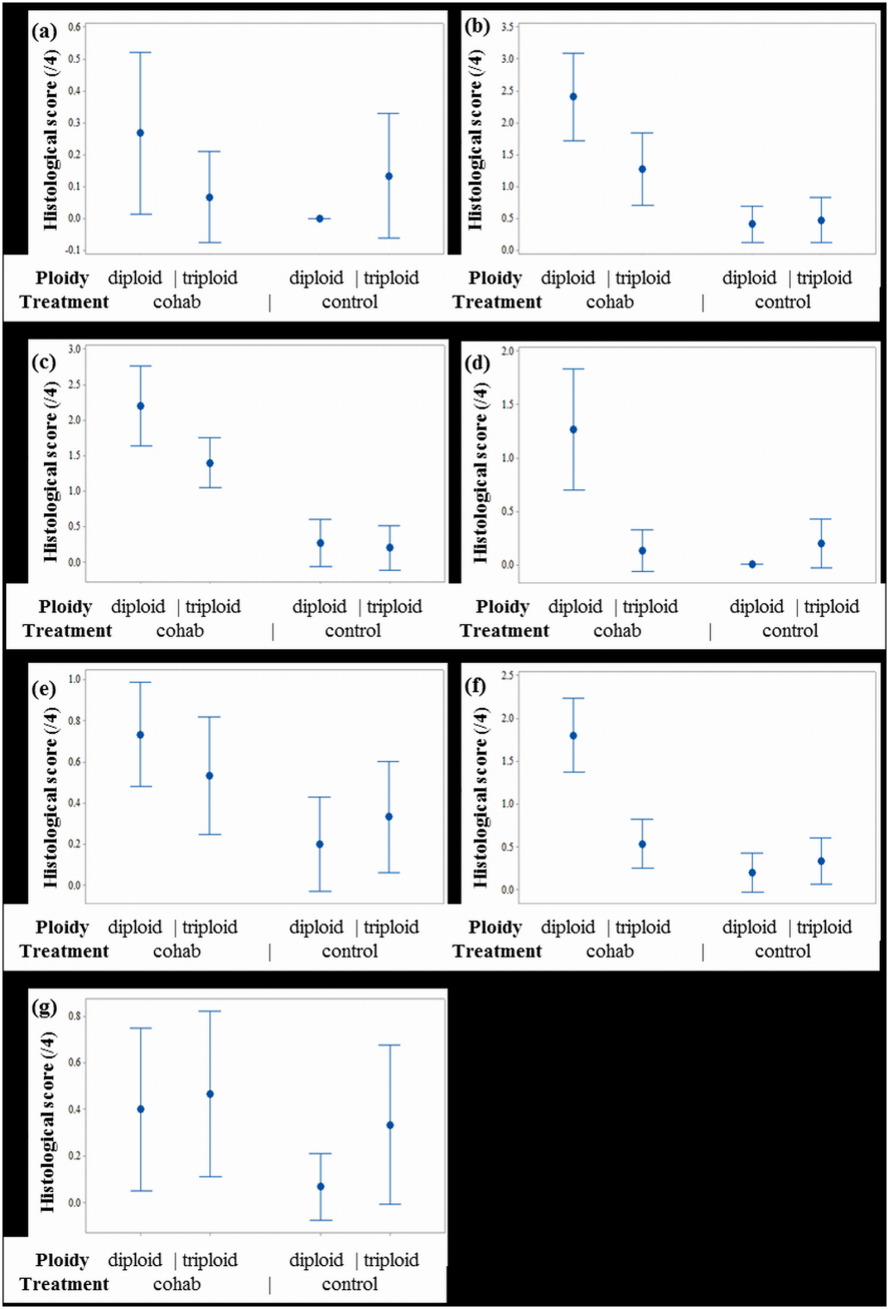
Interval plots comparing average histological scores between ploidy and treatment. (a) Liver inflammation, no significant differences detected between ploidy or treatments; (b) Liver degeneration. Significant difference between treatments; (c) Pancreas inflammation. Significant difference between treatments; (d) Pancreas degeneration. Significant difference between ploidy (more severe degeneration in diploids) and treatment; (e) Heart inflammation. Significant difference between treatments; (f) Heart degeneration. Significant difference between ploidy (more severe degeneration in diploids) and treatment (g), Epicarditis. No significant difference between ploidy or treatment.

The difference in the histopathology scores between diploids and triploids as a result of the SAV infection was only assessed across the co-hab group. The histology scoring results revealed, degree of heart inflammation, heart degeneration, pancreas inflammation, pancreas degeneration and liver degeneration were significantly different between the co-hab and the control fish (Fig 7).

With respect to differences between ploidies, the only significant difference detected was in the degree of heart degeneration, in which diploids has a significantly higher degree of degeneration compared to triploids. The cross-checked with the cumulative link model, factoring in the random effect of replicate tanks found a significant difference between ploidy with regards to pancreatic degeneration (p = 0.046).

Moreover, there were significant differences between certain tanks in the degree of liver and pancreatic degeneration. However, no any statistically significant differences were detected in skeletal muscle or kidney lesion scores between any of the groups. When the tank effect was evaluated using a cumulative link model and likelihood ratio test, it was not found to have influenced the analytical results, but revealed a significant effect of ploidy on pancreas degeneration, which was not detected using a Kruskal-Wallis test. Principal component analysis of heart, pancreas and liver scores at the tank level showed that 85.2% of variance was explained by a single component along, which all variables were weighted. Liver degeneration had the strongest loading along this component (-0.562) and epicarditis and liver inflammation the weakest loadings.

## Discussion

This study was performed to examine the susceptibility and pattern of disease pathogenesis in diploid and triploid Atlantic salmon fry exposed to SAV1 through an IP, immersion or co-habitation route of infection. Cano and others [26] recently established an SAV1 infection in Atlantic salmon fry using an immersion route of infection. The present study is the first report of a SAV infection in both triploids and diploids using IP and co-habitation routes to infect the fish. Lesions of SAV infection were observed primarily in the pancreas and heart of infected fry, but were not pronounced in skeletal muscle. Degenerative lesions were seen in the liver and kidney of some groups however, which are not usually observed during experimentally-induced or naturally occurring clinical SAV infections of older fish [12, 15]. The differences of pathological presentation seen in the fry in the present study was similar to that observed by Cano and others [26] compared to previous experimental and clinical infections in adult fish [15], and may be indicative of fry being less active or having lower levels of immune competence.

Salmonid alphavirus infection is a disease that occurs after fish are transferred to seawater [27], and which can also be reproduced experimentally [28]. SAV infection has been studied in Atlantic salmon parr and pre-smolts in fresh water, rainbow trout reared in fresh water and in Atlantic salmon smolts in sea water [17]. The mortalities experienced during experimental infections with SAV1 are highly variable and unpredictable [12], although the average level of mortality seen in the present study was within the reported range for mortalities during a natural outbreak of PD [16]. Nonetheless, the levels of mortality that occurred in the fry were above those generally expected for an experimental SAV1 infection [29,30] possibly due to immune-incompetence in fry of the size used in the present study. Furthermore, the severity and prevalence of histological lesions resulting from SAV1 infection have been shown to vary significantly between strains of salmon [29], supporting theories from field observations that certain farmed strains are more susceptible to SAV1 infection than others [29]. This must be taken into consideration when comparing results between SAV1 experiments.

The significantly higher mortality rate observed following IP administration of the virus compared to the other routes of exposure is expected given the more invasive, direct inoculation of a high pathogen load, bypassing the natural barriers of infection [30]. Nevertheless, the cumulative mortalities of IP-challenged fish in this trial clearly highlighted a lack of significant difference in susceptibility between diploid and triploid Atlantic salmon to an experimental SAV1 infection when challenged via this route.

The diploid fry in both the co-hab and IM groups had significantly higher levels of SAV RNA in their liver tissue. In heart samples, however, the levels of SAV RNA detected in diploid hearts were lower compared to their triploid hearts in all groups. This may contradict the widely held belief that diploid salmon are generally more disease resistant than triploids [2]. Most of the pathogen challenges used to compare disease susceptibility between diploid and triploid fish have focused on extracellular pathogens [7], which do not directly use host cell metabolic machinery for replication. Since transcription efficiencies appear to show a dose effect, however [7], triploid and diploid fish may not prove similar under a viral pathogen challenge, resulting in lower virus yield in the triploids compared to diploids, as seen in the present study.

The nature and distribution of histopathological lesions observed between the two ploidies were generally comparable, further supporting the hypothesis that SAV 1 affects diploid and triploid fish to a similar extent. When a more objective approach was used, implementing the scoring system to quantify levels of pathology, both heart and pancreatic degeneration was found to be significantly worse in diploid fry in the cohabitation group. Mild degenerative changes and inflammatory response was observed in some of the control fry, and moderate epicarditis was also seen in one control individual. Epicarditis in Atlantic salmon is generally considered a non-specific response however, and therefore observation of mild epicarditis observed in the present study is considered to be a normal finding [24]. Furthermore, as a score of 1 was given when even a single degenerate cell was observed, it is not surprising that a number of fish had scores above zero even in the absence of disease. Indeed, at least two out of the five fry from each control tank scored 1 in at least one of the tissues evaluated, and it is therefore not unreasonable to consider a score of 1 in the context of this study as normal, as previously described by other authors [24]. The number of control fry with the presence of histological changes of possible significance (score 2 or higher) in any of the tissues screened was 4 out of the 30 fry examined. This is in direct contrast to the virus-challenged fry, in which 27 fry scored a 2 or higher in at least one of their tissues.

The severe degenerative changes observed in the heart of some SAV1 exposed fry, particularly individuals from the IP and cohabitation tanks, were similar to those described previously [31,32], whilst other fry displayed less severe lesions were more in line with findings reported by other authors [12]. Observed pancreatic lesions, ranging from acute focal acinar necrosis to complete absence of acinar tissue, were similar to those previously described by [21] for fish in acute/sub-acute phases of SAV1 infection.

McLoughlin and Graham [15] reported that skeletal muscle lesions are not typically seen until 3–4 weeks after the development of pancreatic and cardiac lesions, whereas samples in this study were collected at 17 dpi. In previous SAV1 investigations involving parr or pre-smolts, muscular lesions were not a typical feature and were mild, possibly due to low physical activity in younger fish [24,28]. Collectively, these earlier findings could explain why skeletal muscle pathology was largely absent in this study.

Hepatic pathology is often associated with primary pancreatic disease in humans and animals [33,34], and it has also been described in association with other viral infections in salmon [35]. Ferguson *et al*. [32] reported multi-focal to focally extensive hepatic necrosis in PD infected fish, whilst a study by Taksdal *et al*. [14] observed diffuse single-cell necrosis or apoptosis in SAV1 infected salmon. Similarly, renal degeneration, as observed in this study, is not commonly considered a pathology associated with PD pathogenesis. Degeneration in the liver and kidneys is also frequently observed as a secondary change in cases of cardiac failure in mammalian species [36,37]. While this is a possible explanation of the pathology reported in the current study, the generalised mild to moderate infiltration of mononuclear cells into the hepatic parenchyma and distribution of hepatic change is not typical of secondary congestive damage [38], thus direct viral induced hepatitis is more probable. McLoughlin and Graham [15] stated that PD is characterised by a spectrum of lesions, however, the precise mechanisms involved in pathogenesis are still not fully understood, despite this being an area that has been more extensively explored in recent years [39–42]. Further investigation, including improved methods for the detection of virus, would help to elucidate which changes in this study were due to direct viral damage and which were secondary sequelae.

Collectively, the results observed in this study agree with other recent findings investigating the health performance of triploid salmonids. For example, Weber *et al*. [41] found similar survival rates between diploid and triploid rainbow trout challenged with *Flavobacterium psychrophilum*. Similarly, a set of trials conducted by Frenzl *et al*. [43] showed no difference in their susceptibility to sea lice infection. In contrast, Ozerov *et al*. [6] found a higher rate of parasitism with *Gyrodactylus salaris* in triploids compared with full-sib diploid Atlantic salmon, however it has been suggested that key infection properties affecting *G*. *salaris* burden, specifically the size of fish, were not taken into consideration in the former study. However, further research into the susceptibility of triploids to SAV1 in the field is necessary to establish whether the results of this study translate to findings under commercial production conditions.

The susceptibility of both diploid and triploid fry to SAV infection was demonstrated in the present study, providing a valid modelfor SAV1 infection in furher experimental studies. This may be of particular use to those investigating salmonid strain differences in SAV1 susceptibility for genetic improvement and vaccine research. Robust scientific field studies investigating how major pathogens affect triploid Atlantic salmon in aquaculture are limited and furthermore, as triploids show higher levels of mortality than diploids during periods of environmental stress [41], studies comparing disease susceptibility between ploidy types under suboptimal environmental conditions are needed to more accurately predict performance of triploid salmon in intensive culture systems. This information is essential in providing more conclusive evidence as to whether ploidy significantly affects disease resistance.

## Supporting information

S1 Fig RT-qPCR result of SAV infected fish. The mean Cp value, mean estimated SAV copy number and infection states; positive (+ ve) and negative(-ve) for each individual fish.
(PDF)

S2 Fig Skeletal muscle pathology. Pathological changes were observed in the intraperitoneally challenged fish. Inflammatory infiltration and degenerative changes were specifically present in the red muscle.
(PDF)

S3 Fig Liver pathology. Severely damaged liver tissues stained with Haematoxylin and Eosin (H&E), periodic acid-schiff (PAS) and anti-human CD3 polyclonal antibody (CD3). The histological presentation suggest an ongoing peroxidative type of tissue damage and also possible virus induced hepatitis.
(PDF)
