# Supplementary material for: Impact of Salmonid alphavirus infection in diploid and triploid Atlantic salmon (Salmo salar L.) fry
Source: PLoS One. 2017 Sep 26;12(9):e0179192. doi: 10.1371/journal.pone.0179192 (PMC5614425; doi:10.1371/journal.pone.0179192)
Supplement: S1 Fig — The mean Cp value, mean estimated SAV copy number and infection states; positive (+ ve) and negative(-ve) for each individual fish. (PDF) [file pone.0179192.s001.pdf]

	Experiment Info			Heart			Liver		
	Sample No.	Route	Tank	Mean Cp	Mean copy no.	SAV +/-	Mean Cp	Mean copy no.	SAV +/-
Diploids	1	Co-hab	T1	18.56	538979.20	+ ve	29.73	17124.57	+ ve
	2	Co-hab	T1	20.89	141221.48	+ ve	24.79	26959.31	+ ve
	3	Co-hab	T1	30.38	648.12	+ ve	30.57	331.06	+ ve
	4	Co-hab	T1	18.74	488588.36	+ ve	26.81	5779.69	+ ve
	5	Co-hab	T1	22.10	69919.58	+ ve	26.44	7628.90	+ ve
	11	Co-hab	T3	28.54	1773.56	+ ve	26.39	6915.38	+ ve
	12	Co-hab	T3	25.05	17874.62	+ ve	32.15	240.89	+ ve
	13	Co-hab	T3	25.12	17138.15	+ ve	29.74	903.93	+ ve
	14	Co-hab	T3	23.73	42648.10	+ ve	27.16	4333.53	+ ve
	15	Co-hab	T3	26.76	5763.51	+ ve	32.35	183.11	+ ve
	21	Co-hab	T5	*	*	- ve	*	*	- ve
	22	Co-hab	T5	*	*	- ve	*	*	- ve
	23	Co-hab	T5	*	*	- ve	*	*	- ve
	24	Co-hab	T5	28.51	273.48	+ ve	26.23	4974.41	+ ve
	25	Co-hab	T5	*	*	- ve	*	*	- ve
	31	IM	T7	30.74	473.44	+ ve	24.15	43610.09	+ ve
	32	IM	T7	21.03	130330.91	+ ve	23.55	69027.21	+ ve
	33	IM	T7	27.40	3269.87	+ ve	*	*	- ve
	34	IM	T7	23.29	35151.63	+ ve	23.81	56441.22	+ ve
	35	IM	T7	22.19	66822.47	+ ve	26.82	5755.46	+ ve
	41	IM	T9	21.67	167795.86	+ ve	27.64	3221.88	+ ve
	42	IM	T9	31.26	291.05	+ ve	*	*	- ve
	43	IM	T9	19.92	535813.43	+ ve	19.31	518671.72	+ ve
	44	IM	T9	30.06	645.35	+ ve	25.16	14586.24	+ ve
	45	IM	T9	29.55	906.75	+ ve	34.93	38.30	+ ve
	51	IM	T11	22.17	18074.61	+ ve	24.69	13308.04	+ ve
	52	IM	T11	27.40	566.50	+ ve	26.85	3598.88	+ ve
	53	IM	T11	26.24	1384.75	+ ve	28.22	1358.49	+ ve
	54	IM	T11	24.31	4573.59	+ ve	29.14	753.59	+ ve
	55	IM	T11	27.83	463.07	+ ve	28.99	928.41	+ ve

	61	IP	T13	22.02	73060.08	+ ve	*	*	- ve
	62	IP	T13	22.68	50719.97	+ ve	*	*	- ve
	63	IP	T13	22.88	44682.39	+ ve	29.38	916.48	+ ve
	64	IP	T13	21.15	140943.18	+ ve	35.47	9.43	+ ve
	65	IP	T13	20.72	173136.20	+ ve	31.06	227.49	+ ve
	71	IP	T15	25.73	11368.41	+ ve	34.08	88.16	+ ve
	72	IP	T15	31.01	344.05	+ ve	30.39	603.40	+ ve
	73	IP	T15	22.64	88067.70	+ ve	32.67	182.39	+ ve
	74	IP	T15	19.08	930264.74	+ ve	26.92	5015.45	+ ve
	75	IP	T15	26.29	7850.18	+ ve	34.69	43.86	+ ve
	81	IP	T17	27.26	620.85	+ ve	31.05	225.39	+ ve
	82	IP	T17	30.65	73.97	+ ve	35.29	15.22	+ ve
	83	IP	T17	28.06	366.65	+ ve	33.94	36.96	+ ve
	84	IP	T17	25.90	1521.46	+ ve	37.47	3.80	+ ve
	85	IP	T17	26.63	1657.26	+ ve	34.28	29.09	+ ve
	6	Co-hab	T2	31.05	409.06	+ ve	40.70	0.33	+ ve
	7	Co-hab	T2	*	*	- ve	30.15	510.12	- ve
	8	Co-hab	T2	32.03	234.95	+ ve	28.03	2414.22	+ ve
	9	Co-hab	T2	22.18	68794.07	+ ve	33.88	27.26	+ ve
	10	Co-hab	T2	31.97	310.53	+ ve	22.87	115427.62	+ ve
	16	Co-hab	T4	30.46	498.14	+ ve	28.80	1585.74	+ ve
	17	Co-hab	T4	26.80	5598.26	+ ve	30.40	598.86	+ ve
	18	Co-hab	T4	23.26	61538.93	+ ve	26.14	8100.70	+ ve
	19	Co-hab	T4	27.00	4894.57	+ ve	26.38	8339.60	+ ve
	20	Co-hab	T4	23.55	48689.45	+ ve	35.43	28.91	+ ve
	26	Co-hab	T6	*	*	- ve	*	*	- ve
	27	Co-hab	T6	*	*	- ve	*	*	- ve
	28	Co-hab	T6	*	*	- ve	*	*	- ve
	29	Co-hab	T6	*	*	- ve	*	*	- ve
	30	Co-hab	T6	*	*	- ve	*	*	- ve
	36	IM	T8	32.47	194.72	+ ve	*	*	- ve
	37	IM	T8	25.79	8594.40	+ ve	37.75	1.44	+ ve
	38	IM	T8	22.34	60571.75	+ ve	28.78	1300.40	+ ve

**Trploids**	39	IM	T8	26.33	6040.80	+ ve	0.00	*	- ve
	40	IM	T8	19.10	397802.85	+ ve	31.02	236.78	+ ve
	46	IM	T10	28.24	2151.78	+ ve	35.06	34.98	+ ve
	47	IM	T10	23.17	61936.33	+ ve	23.82	33538.85	+ ve
	48	IM	T10	*	*	- ve	35.59	25.33	+ ve
	49	IM	T10	35.84	13.92	+ ve	*	*	- ve
	50	IM	T10	29.63	856.72	+ ve	*	*	- ve
	56	IM	T12	24.79	3803.73	+ ve	24.64	13204.15	+ ve
	57	IM	T12	24.24	5116.88	+ ve	29.21	841.78	+ ve
	58	IM	T12	20.80	44451.87	+ ve	22.78	53444.34	+ ve
	59	IM	T12	29.98	104.42	+ ve	33.76	42.06	+ ve
	60	IM	T12	30.09	96.62	+ ve	25.90	7350.83	+ ve
	66	IP	T14	31.38	349.65	+ ve	32.50	80.40	+ ve
	67	IP	T14	*	*	- ve	35.44	8.59	- ve
	68	IP	T14	30.27	620.71	+ ve	29.42	866.07	+ ve
	69	IP	T14	*	*	- ve	39.33	0.48	- ve
	70	IP	T14	21.85	80571.39	+ ve	32.35	88.99	+ ve
	76	IP	T16	28.25	2139.83	+ ve	33.17	113.43	+ ve
	77	IP	T16	22.09	127073.36	+ ve	27.23	4138.05	+ ve
	78	IP	T16	21.98	137079.41	+ ve	36.21	18.07	+ ve
	79	IP	T16	17.94	1988080.81	+ ve	33.18	110.51	+ ve
	80	IP	T16	33.11	85.27	+ ve	34.33	59.40	+ ve
	86	IP	T18	28.11	380.66	+ ve	32.95	90.15	+ ve
	87	IP	T18	23.48	7579.52	+ ve	30.86	258.35	+ ve
	88	IP	T18	28.04	406.70	+ ve	32.80	136.86	+ ve
	89	IP	T18	31.86	30.85	+ ve	34.34	27.81	+ ve
	90	IP	T18	30.18	116.79	+ ve	33.45	48.83	+ ve
