# Supplementary material for: Impact of Salmonid alphavirus infection in diploid and triploid Atlantic salmon (Salmo salar L.) fry
Source: PLoS One. 2017 Sep 26;12(9):e0179192. doi: 10.1371/journal.pone.0179192 (PMC5614425; doi:10.1371/journal.pone.0179192)
Supplement: S2 Fig — Pathological changes were observed in the intraperitoneally challenged fish. Inflammatory infiltration and degenerative changes were specifically present in the red muscle. (PDF) [file pone.0179192.s002.pdf]

One fish in IP challenged group;

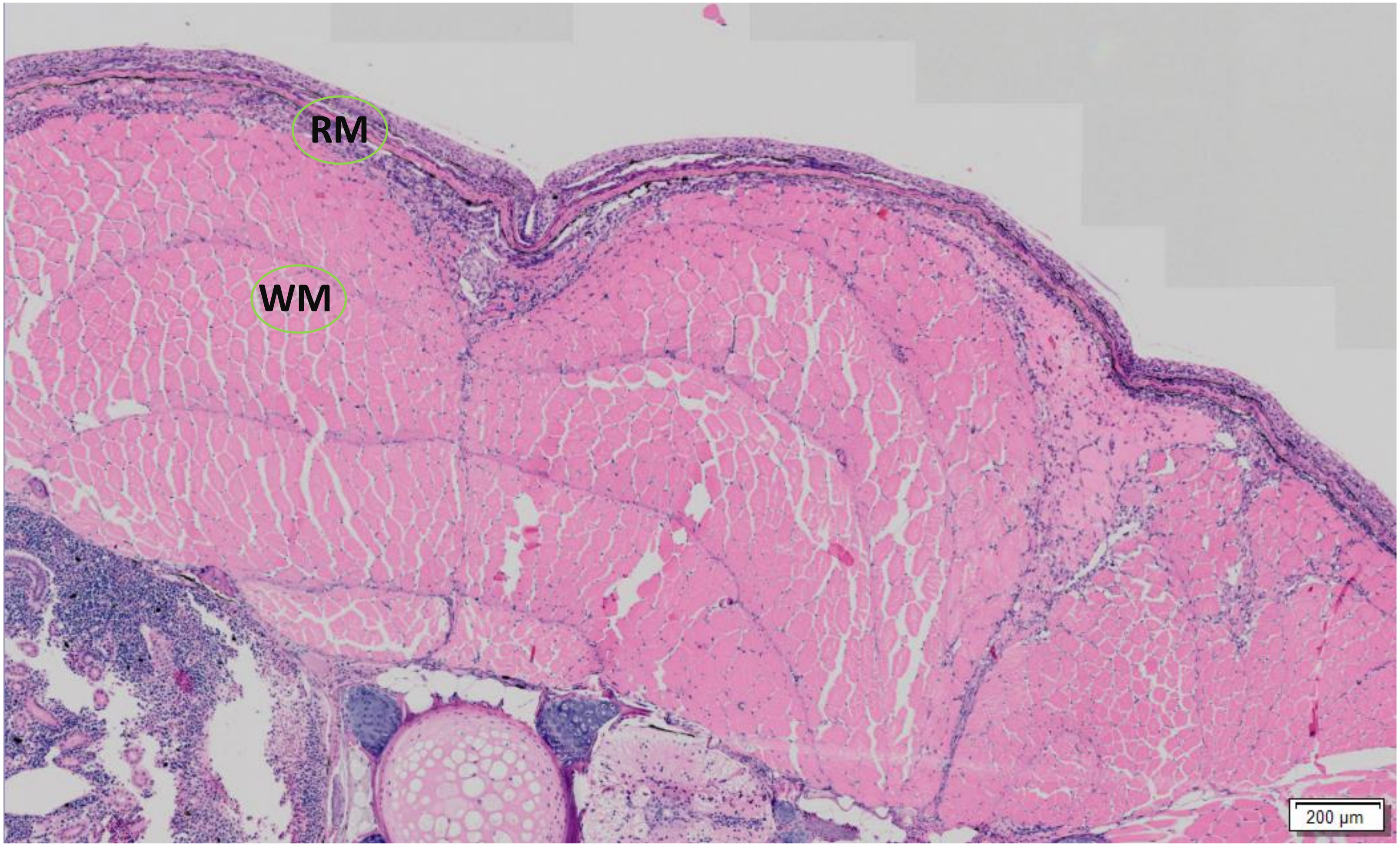

Red muscle (RM) – sever diffuse necrosis and inflammation
White muscle (WM) - moderate multifocal necrosis and inflammation
