# Supplementary figures and images for: Impact of Salmonid alphavirus infection in diploid and triploid Atlantic salmon (Salmo salar L.) fry
Source: PLoS One. 2017 Sep 26;12(9):e0179192. doi: 10.1371/journal.pone.0179192 (PMC5614425; doi:10.1371/journal.pone.0179192)

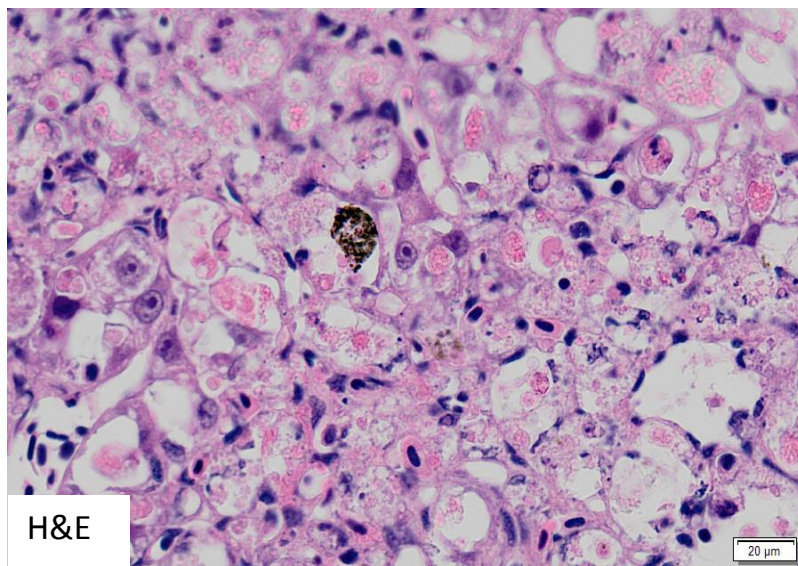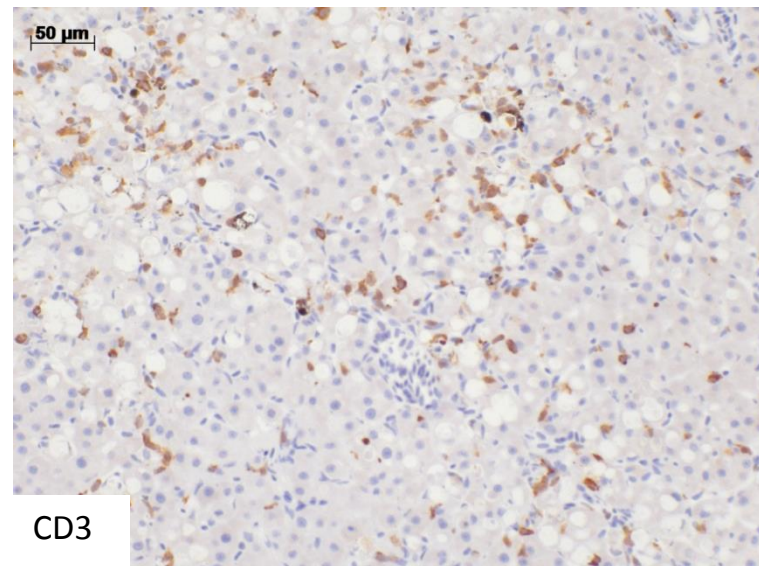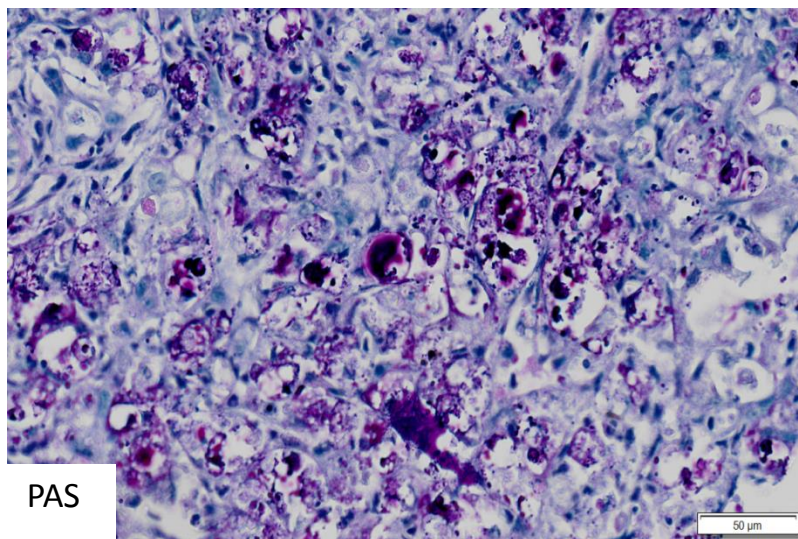

- Peroxidative type of damage
- Hepatitis (virus or ROX ?)

**Severe diffuse viral hepatitis**

Supplement: S3 Fig — Severely damaged liver tissues stained with Haematoxylin and Eosin (H&E), periodic acid-schiff (PAS) and anti-human CD3 polyclonal antibody (CD3). The histological presentation suggest an ongoing peroxidative type of tissue damage and also possible virus induced hepatitis. (PDF) [file pone.0179192.s003.pdf]
